# A survey on the prevalence of diarrhea in a Portuguese population of police working dogs

**DOI:** 10.1186/s12917-021-02920-y

**Published:** 2021-06-07

**Authors:** J. C. Alves, P. Jorge, A. Santos

**Affiliations:** 1 Divisão de Medicina Veterinária, Guarda Nacional Republicana (GNR), Rua Presidente Arriaga, 9, 1200-771 Lisbon, Portugal; 2 MED – Mediterranean Institute for Agriculture, Environment and Development, Instituto de Investigação e Formação Avançada, Universidade de Évora, Pólo da Mitra, Ap. 94, 7006-554 Évora, Portugal

**Keywords:** Working dog, Diarrhea, Activity level

## Abstract

**Background:**

Diarrhea is considered the most common clinical sign of chronic gastrointestinal disease in dogs and affects a considerable portion of working and sporting dogs. We aimed to determine the prevalence of diarrhea in police working dogs and evaluate the relationship between feeding, activity level, and animal characteristics with clinical signs. In an observational, prospective study, information on 188 dogs was collected. For each patient, age, sex, breed, specific mission, number of animals at the same housing location, and activity level was recorded. A body condition (BCS) and canine inflammatory bowel disease activity index (CIBDAI) scores were determined, and feces classified according to the Bristol Stool Form Scale. The Kruskal-Wallis test was used to compare recorded data between breeds, mission, age, and sex. Multiple regression was run to predict BCS score, increased defecation frequency, diarrhea, CIBDAI scores, Bristol stool scores, diarrhea from activity level, number of animals at the same housing location, breed, and mission. A *p* < 0.05 was set.

**Results:**

Animals in the sample (male *n* = 96, female *n* = 92) had a mean age of 5.2 ± 3.2 years and a bodyweight of 24.1 ± 7.2 kg. Four main dog breeds were represented, 80 Belgian Malinois Shepherd Dogs, 52 German Shepherd Dogs, 25 Labrador Retrievers, and 19 Dutch Shepherd Dog. A prevalence of diarrhea of 10.6% was determined, with 4% of dogs having liquid diarrhea. Dogs classified as “extremely active” were more likely to have a low BCS, and the level of activity contributed to diarrhea and BCS prediction.

**Conclusion:**

Police working dogs frequently experience diarrhea episodes, which lead to clinical disease and performance loss. Investigation of aetiologies is required.

## Background

Police forces employ dogs throughout the world in various tasks, including product detection (drugs, explosives, or others), protection, and search and rescue, amongst others [1]. Diarrhea, defined as an increase in fecal water content usually leading to changes in fecal volume, fluidity, and frequency of defecation [2, 3], is a common clinical sign of chronic inflammatory enteropathies, considered the most common cause of chronic gastrointestinal disease in dogs [4]. Diarrhea affects a considerable portion of working and sporting dogs [5, 6], with a reported prevalence of 7.5–36% in long-distance racing sled dogs. However, it is thought to occur with much greater frequency, representing a leading cause of morbidity in racing sled dogs, causing some performance loss [5]. A possible cause for diarrhea in these animals may be the effect of prolonged exercise on the gastrointestinal tract [5]. A similar condition is described in human athletes, referred to as runner’s diarrhea is an acute exercise-induced diarrhea [7].

This condition is usually present with unremarkable complete blood cell count, serum biochemistry, and fecal examinations [8]. The physical examination can be normal or, for some animals, reveal secondary clinical signs such as weight loss, abdominal pain, and flatulence [4]. As in humans athletes, it is crucial to identify and manage the effect that training, lifestyle stresses, and dietary practices [9] can have on working and sporting dogs and the occurrence of diarrhea and other clinical signs [10].

The canine inflammatory bowel disease Index (CIBDAI) can be used to follow patients and assess response to treatment. It is a reliable measure of inflammatory activity in dogs [11]. Recording other clinical signs, such as feces characteristics and bodyweight variation, is also important [4]. The Bristol fecal chart can aid fecal scoring. Although it has not been validated in dogs, it has been in humans and extensively utilized in humans [12], showing substantial validity and reliability [13].

This study’s objective was to determine the prevalence of diarrhea in police working dogs and evaluate the relation between feeding, activity level, and animal characteristics, with clinical signs. We hypothesized that these factors influence clinical signs.

## Results

Information on 188 animals was collected. Dogs in this sample had a mean age of 5.2 ± 3.2 years and a bodyweight of 24.1 ± 7.2 kg, representing both sexes (male *n* = 96, female *n* = 92). Four main dog breeds were represented, 80 Belgian Malinois Shepherd Dogs, 52 German Shepherd Dogs, 25 Labrador Retrievers, 19 Dutch Shepherd Dog, and 12 animals from other breeds. Regarding specific missions, 83 (44%) animals were use of force dogs, 73 (39%) were product detection dogs (drugs or explosives), and 32 (17%) were search and rescue dogs. When the handlers were asked to classify their dog’s activity level, 24 (13%) were classified as “calm”, 121 (65%) as “active”, and 43 (22%) as “extremely active”.

All animals were fed throughout the day, mainly during training, as a part of a positive reinforcement program. One hundred and twenty-eight animals (68%) were fed 2 times/day, 55 (29%) were fed 3 times/day, and 5 (3%) were fed 4 times/day. Mean FBR was 20.2 ± 7.3 g/kg.

Regarding defecation frequency, 23 (12%) animals had an increased frequency (21, 11% - 4x/day, and 2, 0.01% - 5x/day), while 73 (39%) had a defecation frequency of 2 x/day, and 92 (48.9%) of 3x/day. According to the Bristol stool form scale, 20 (10.6%) had a grade corresponding to diarrhea, divided between grades 5 (*n* = 8), 6 (n = 8), and 7 (*n* = 4). Animals without diarrhea had faeces graded as 2 (*n* = 55), 3 (*n* = 85) and 4 (*n* = 28). A majority of animals had low CIBDAI scores, with 159 (84.5%) dogs scoring at the level of clinically insignificant disease. For 14 animals (15.5%), a score corresponding to mild disease (4 or 5) was recorded.

A significant difference was observed in activity level (*p* = 0.02) and body condition score (BCS) (*p* = 0.03), considering the comparison between breeds. Labrador Retriever recorded more classifications of “calm” for activity level, while Belgian Malinois and Dutch Shepherd Dog had more classifications of “extremely active”. These two breeds (Belgian Malinois and Dutch Shepherd Dog) also had lower BCS scores. No significant differences were observed between specific missions, age, and sex. BCS was significantly predicted by activity level F(1, 186) = 20.746, *p* < 0.01, R2 = 0.096, Bristol score F(1, 186) = 10.313, p < 0.01, R2 = 0.047, and CIBDAI score F(1, 186) = 10.157, p < 0.01, R2 = 0.052. The frequency of defecation was predicted by Bristol stool score F(1, 186) = 6.234, *p* = 0.01, R2 = 0.027, and the classification of feces according to the Bristol stool chart was predicted by activity level F(1, 186) = 14.149, *p* = 0.02, R2 = 0.103, and the number of animals in the same location F(1, 186) = 3.177, *p* = 0.01, R2 = 0.132. On the other hand, a stool score corresponding to diarrhea was predicted by activity level F(3, 91) = 1.029, *p* = 0.02, R2 = 0.086, and FBR F(3, 91) = 7.918, p = 0.02, R2 = 0.086. CIBDAI scores were significantly predicted by FBR F(1, 91) = 1.560, *p* = 0.04, R2 = 0.018.

## Discussion

This study assessed the prevalence of diarrhea in police working dogs and its relation with feeding, activity level, and animal characteristics in a relatively large cohort of dogs. According to the Bristol stool chart grade, the prevalence of diarrhea was 10.6%, with 4% of dogs having liquid diarrhea. A similar number was observed if increased defecation frequency (12%).

Other canine athletes, like sled dogs, show a high prevalence of diarrhea (36%) during athletic events. This high prevalence was led researchers to investigate the etiology, with limited results [14]. It is known that stress and exercise can induce episodes of diarrhea [6]. Stress is defined as an external disturbance or threat from the environment that disturbs homeostasis, and in rodent models, chronic stress induces abnormalities in small and large intestines [8]. Sporting and working events cause mental and physical stress and is the prevailing theory for the diarrhea episodes observed in racing sled dogs [14]. Similar effects are observed in human athletes, with GI symptoms associated with various factors, including the type and duration of exercise [15]. We did not observe significant differences between specific missions. While the amount of physical effort varies significantly between different missions (comparing a drug detection to a search and rescue dog, for example) [16], all of the dogs included in the sample go through a similar physical conditioning program [17], which may account for this lack of differences. A strong association was observed between racing and the presence of diarrhea, blood, or mucus in the feces, in up to 46.5% of dogs studied has been reported in one report [5]. We evaluated the presence of diarrhea during the entire set of activities and rest periods, not during active work per si, which may also account for some registered differences. Still, the management of diarrhea or diarrhea episodes is something to keep in mind for assisting veterinarians, as it will undoubtedly have a toll on overall performance [5].

Stress is an important initiating cause of diarrhea, particularly in working dogs [18]. Kenneling is also a primary factor, which increases the likelihood of diarrhea episodes [2]. The intestinal tract is highly susceptible to stress, and clinical signs can develop rapidly and last for long periods [19]. Despite this relationship between stress, personality traits, and personality traits, there is no temporal relationship between them and the beginning of clinical signs [20]. We sought to evaluate this effect by determining the relationship between clinical signs and described activity levels, breed, and the number of animals at the same housing location. Belgian Malinois and Dutch Shepherd Dog had significantly higher activity levels and lower BCS. These two breeds and Belgian Malinois are usually described as and selected based on very high drive, which can be desirable for working dogs [1]. On the other hand, it may turn them more prone to episodes of diarrhea, as a stool score corresponding to diarrhea was predicted by activity level. The management of stress in working dogs from a very early age has recently increased interest [21, 22] and may help address this issue.

Stool form scales are a standardized and accessible method of classifying stool form by all involved in patient care, improving communication between veterinarians and the owner [13]. Even though fecal scoring is subjective, with some intra-observer and interobserver variability [2], the Bristol fecal chart appears to perform adequately in dogs [12]. Still, some level of incorrect classification may have occurred, particularly between grades 4 and 5, which set the limits of the classification as diarrhea or not. Higher categories in the Bristol fecal chart predicted the frequency of defecation. According to the Bristol stool chart, the type of faces was predicted by activity and the number of animals in the same housing location. It is known that the breed of a dog affects digestion, with larger breeds typically having less ability to digest foods [23], and German Shepherd Dogs, for example, appear predisposed to antibiotic responsive enteropathies [24]. We did observe significant differences between breeds, particularly in activity levels and BCS scores. Activity levels may influence diarrhea development that, left untreated, can lead to a poor BCS. A higher number of animals in the same housing location can also lead to higher environmental stress, adding to this overall mechanism.

In contrast to Bristol stool chart classifications, CIBDAI scores were only significantly predicted by FBR. The relationship between CIBDAI and FBR is unclear. Overfeeding can lead to diarrhea [25], and dogs being fed higher amounts of food may develop diarrhea which, left uncontrolled, can produce high CIBDAI scores. It is also possible that patients will high CIBDAI scores are overfed due to showing body weight and BCS losses. Probably, a combination of the two is present, but future studies should explore this.

The study presents some limitations: only the prevalence of diarrhea was investigated and not its etiology. More than one source may be present, as it is a common clinical sign with different causes. Aditional possible etiologies should be explored in future studies. Also, the feces type at the moment of the survey was determined, and some animals may show intermittent diarrhea, which will cause an underestimation of diarrhea’s prevalence. Even though handlers were requested to answer the questions honestly, some bias may occur, particularly in cases that may be considered unfavorable, as events of diarrhea. Future studies are also warranted to compare these findings with different populations of police working dogs.

## Conclusions

This study showed a prevalence of diarrhea of 10.6% according to the Bristol stool chart grade, with 4% of dogs having liquid diarrhea. No significant differences were observed between specific missions, age, and sex. However, Belgian Malinois and Dutch Shepherd Dog were more frequently classified as “extremely active”, and dogs with this classification were more likely to have a low BCS. The occurrence of diarrhea was significantly predicted by activity level and FBR.

## Methods

In this observational, prospective study, active police working dogs of the Guarda Nacional Republicana were screened (Republican National Guard Canine Unit, Portugal). To be included in the study, animals should be active police working dogs, have a bodyweight ≥15 kg, and an age of 1.5–9 years. All patients were regularly vaccinated and dewormed (the last occurring 1–2 months before this survey). They were kept in kennels of the Guarda Nacional Republicana, similar in size and construction, in locations spread through Portugal’s territory.

For each patient, the name of the handler, dog name, age closest to the year, sex, breed, specific mission, and the number of animals at the same housing location was recorded. Handlers were asked to classify the level of spontaneous activity of their dogs as “calm”, “active”, or “extremely active”. The frequency and amount of feeding administered were recorded. A food amount (in grams)/body weight (in kilograms) ratio (FBR) was calculated to compare the given food amount between different sized dogs. All dogs were fed the same commercially available dog food (HappyOne High Energy, petMaxi, Portugal), with 28% protein, 15% fat, 8% minerals, and 2.75% fiber. No additional treats were provided. Additionally, a body condition score [26] and CIBDAI scores [27] were determined, and feces classified according to the Bristol Stool Form Scale demonstrated substantial validity and reliability [13]. Fecal scores of ≤4 considered were considered to be non-diarrheic [3, 12]. The mean number of defecations per day was also recorded [28], and a number of defecation > 3 was deemed abnormal.

Normality was assessed with a Shapiro-Wilk test. The Kruskal-Wallis test was used to compare activity level, BCS scores, defecation frequency, the presence of diarrhea, Bristol stool scores, and CIBDAI score between breeds, mission, age, and sex. Multiple regression was run to predict BCS, increased defecation frequency, diarrhea, CIBDAI score, Bristol stool scores, and diarrhea from FBR, activity level, number of animals at the same housing location, breed, and mission. All results were analyzed with IBM SPSS Statistics version 20, and a significance level of *p* < 0.05 was set.

## Data Availability

The datasets used and/or analyzed during the current study are available from the corresponding author on reasonable request.

## Abbreviations

BCS: Body condition score
CIBDAI: canine inflammatory bowel disease index
FBR: Food intake/body weight ratio
